# Editorial Announcement

**Published:** 1914-05

**Authors:** 


**Editorial Announcement.** The April issue was delayed a few days in mailing, for reasons that could not be prevented. Owing to a change of printer, the present issue will probably also be a few days late, and it is possible that some errors may occur until the work of printing is again systematized. A considerable number of plates used to illustrate articles in the JOURNAL have come to light, some of them several years old. These will be held for authors who may care to claim them, until June 1, when they wil be destroyed.

